# Case in Which Two Small Cysts Were Found in the Right Auricle

**Published:** 1826-09

**Authors:** 


*Case in which two small Cysts were found in the right Auricle.*

The Editor was present at the examination of a body at the Edinburgh Infirmary, in 1812, by the late Dr. John Gordon, in which two cysts were found in the right auricle of the heart, in a middle-aged man. One was flat, and applied over the auriculo-ventricular orifice; the other was of a globular form, and about the size of a common marble. Both appeared to consist of concentric layers of organised lymph, about the thickness of a half-crown piece: the globular one was filled with a dark-reddish fluid; the other was empty, but appeared as if there had formerly been a cavity in it. These must have caused great impediment to the circulation, as, on the contraction of the auricle, they must have produced an effect somewhat similar to that of the common globular valve.

The preceding case may, perhaps, throw some light on the manner in which such bodies are formed; as it is probable that, in this case, the cysts were originally in contact with the surface of the auricle, and had become detached, from the motion of the parts.

